# Association and Interaction Analyses of *GABBR1* and *GABBR2* with Nicotine Dependence in European- and African-American Populations

**DOI:** 10.1371/journal.pone.0007055

**Published:** 2009-09-18

**Authors:** Ming D. Li, Jamie E. Mangold, Chamindi Seneviratne, Guo-Bo Chen, Jennie Z. Ma, Xiang-Yang Lou, Thomas J. Payne

**Affiliations:** 1 Department of Psychiatry and Neurobehavioral Sciences, University of Virginia, Charlottesville, Virginia, United States of America; 2 ACT Center for Tobacco Treatment, Education and Research, Department of Otolaryngology and Communicative Sciences, University of Mississippi Medical Center, Jackson, Mississippi, United States of America; 3 Department of Public Health Sciences, University of Virginia, Charlottesville, Virginia, United States of America; Hospital Vall d'Hebron, Spain

## Abstract

Previous studies have demonstrated that the γ-aminobutyric acid type B (GABA_B_) receptor plays an essential role in modulating neurotransmitter release and regulating the activity of ion channels and adenyl cyclase. However, whether the naturally occurring polymorphisms in the two GABA_B_ receptor subunit genes interact with each other to alter susceptibility to nicotine dependence (ND) remains largely unknown. In this study, we genotyped 5 and 33 single nucleotide polymorphisms (SNPs) for GABA_B_ receptor subunit 1 and 2 genes (*GABBR1, GABBR2*), respectively, in a sample of 2037 individuals from 602 nuclear families of African- American (AA) or European-American (EA) origin. We conducted association analyses to determine (1) the association of each subunit gene with ND at both the individual SNP and haplotype levels and (2) the collective effect(s) of SNPs in both GABA_B_ subunits on the development of ND. Several individual SNPs and haplotypes in *GABBR2* were significantly associated with ND in both ethnic samples. Two haplotypes in AAs and one haplotype in EAs showed a protective effect against ND, whilst two other haplotypes in AAs and three haplotypes in EAs showed a risk effect for developing ND. Interestingly, these significant haplotypes were confined to two regions of *GABBR2* in the AA and EA samples. Additionally, we found two minor haplotypes in *GABBR1* to be positively associated with Heaviness of Smoking Index (HSI) in the EA sample. Finally, we demonstrated the presence of epistasis between *GABBR1* and *GABBR2* for developing ND. The variants of *GABBR1* and *GABBR2* are significantly associated with ND, and the involvement of *GABBR1* is most likely through its interaction with *GABBR2*, whereas *GABBR2* polymorphisms directly alter susceptibility to ND. Future studies are needed with more dense SNP coverage of *GABBR1* and *GABBR2* to verify the epistatic effects of the two subunit genes.

## Introduction

Tobacco smoking is a serious public health concern worldwide, as nearly a third of adults smoke tobacco or related products [1]. Nicotine is the main psychoactive substance in cigarettes that functions as a reward and maintains its continued use, ultimately leading to dependence [1]. Meta-analysis of twin and family studies reveals genetic susceptibility to nicotine dependence (ND), with an average heritability of 0.56 [2]. ND also is influenced by environmental factors, as well as by interaction between genetic and environmental factors [2], [3].

Substantial efforts have been geared toward identifying genes that predispose individuals to become nicotine dependent. Genome-wide linkage scans of various smoking phenotypes have revealed several regions that likely harbor susceptibility loci for ND [4], [5], particularly on chromosomes 9, 10, 11, and 17 [4]. Of these reproducibly identified regions, that on chromosome 9 is of particular interest [6], [7], [8], [9], [10]. The first gene identified from this linkage region was *GABBR2* (G-protein coupled receptor 51), for which several SNPs were found to be significantly associated with ND in the Mid-South Tobacco Family (MSTF) cohort [11]. Since that initial report, continued recruitment has yielded approximately a two-fold increase in the sample size of this cohort [10], [12].

GABA is the main inhibitory neurotransmitter in the central nervous system, whose actions are mediated by both ionotropic GABA_A_ receptors and metabotropic GABA_B_ receptors. GABA_B_ receptors are seven transmembrane G-protein-coupled proteins that are pharmacologically functional only as a heterodimer consisting of both GABA_B1_ and GABA_B2_ subunits [13]. GABA neurons are part of the mesolimbic dopamine system, critically important in mediating the reinforcing properties of drugs of abuse. GABA_B_ receptors, in particular, are responsible for dampening the reinforcing effects of dopamine resulting from natural reward. Additionally, the GABA system is diffusely expressed in the brain; therefore, areas other than the mesolimbic system may be partly responsible for these effects. Evidence exists from both animal and human studies supporting the value of GABA_B_ receptor agonists in the treatment of drug abuse. Specifically, in preclinical studies, baclofen, a GABA_B_ agonist, has been successful in promoting abstinence and decreasing the use of several drugs of abuse, including nicotine [14]. Baclofen also has been effective in reducing cigarette smoking [15] and has been reported to alter the sensory properties of cigarettes, reducing their desirability [15].

A recent animal study examined the effect of nicotine on *GABBR2* expression in various brain regions in a rodent model [16]. After chronic nicotine administration, *GABBR2* mRNA was significantly regulated in several brain regions generally associated with addiction, thus providing further evidence that the GABA system is involved in addictive processes. In the mammalian brain, GABA is a major inhibitory neurotransmitter whose modulatory actions are mediated through two types of receptors: the ionotropic GABA_A_ and the metabotropic GABA_B_ [13]. GABA_A_ receptors form ion channels, whereas GABA_B_ receptors activate second-messenger systems thorough G-protein binding and activation. GABA_B_ receptors have two subunits, GABA_B1_ and GABA_B2_, which must form a heterodimer to be pharmacologically active [17]. Thus, the genes that encode these receptor subunits were of particular interest in the current study.

On the basis of previous human and animal studies suggesting that *GABBR2* is involved in the etiology of ND, the current study examined 33 SNPs in *GABBR2* in a larger cohort of the MSTF sample. Because the GABA_B2_ subunit protein does not bind with the neurotransmitter GABA, it must form a heterodimer with the GABA-binding GABA_B1_ subunit [13]; thus, we also examined the association of five SNPs in *GABBR1* with ND.

We found that several SNPs and haplotypes of *GABBR2* were significantly associated with ND in both European-American (EA) and African-American (AA) samples. Although we found no evidence for significant associations of *GABBR1* with ND in either ethnic sample, we detected a significant gene-gene interactive (epistatic) effect between the two subunits by using a newly developed pedigree-based generalized multifactor dimensionality reduction (PGMDR) approach.

## Materials and Methods

### Ethics Statement

Informed written consent was obtained in advance from all participants. The study was approved by the Institutional Review Boards of University of Virginia and University of Mississippi Medical Center and was in accordance with the principles of the Helsinki Declaration II.

### Study Participants

The AA and EA participants were recruited from the US Mid-South States during 1999–2004 [10], [12], [18]. Detailed information on the clinical characteristics of the samples is given in Table 1 and in previous publications from this group [10], [12], [18], [19]. Proband smokers were required to be at least 21 years old, to have smoked for at least the last five years, and to have consumed at least 20 cigarettes per day for the preceding 12 months. After a smoker proband was identified, we attempted to recruit the biological parents and all full siblings. A total of 2037 participants were included in the current study, with 1366 individuals from 402 AA families and 671 individuals from 200 EA families. The average family size (± standard deviation; SD) was 3.14±0.75 for AAs and 3.17±0.69 for EAs. The average age was 39.4±14.4 years for AAs and 40.5±15.5 years for EAs. The ND of probands and other smoker participants was assessed with the three measures commonly used in the tobacco research field: Smoking Quantity (SQ; number of cigarettes smoked per day), the Heaviness of Smoking Index (HSI; 0–6 scale), and the Fagerström Test for Nicotine Dependence score (FTND; 0–10 scale) as a continuous variable. Given the overlap in the content of the three ND measures, there exist fairly robust correlations among them (r = 0.88–0.94) in both the AA and EA samples.

**Table 1:** Clinical characteristics of the African-American, European-American, and pooled samples.

Characteristic	African-Americans	European-Americans	Pooled
No. of nuclear families	402	200	602
Avg. members/family	3.14±0.75	3.17±0.69	3.15±0.73
No. of subjects	1,366	671	2,037
Female (%)	66.1	69.5	67.2
Age (years)	39.4±14.4	40.5±15.5	39.7±14.8
No. of smokers	1,053	515	1,568
Age of smoking onset	17.3±4.7	15.5±4.4	16.7±4.7
Years smoked	20.4±12.5	23.2±13.5	21.3±12.9
Smoking quantity/day	19.4±13.3	19.5±13.4	19.5±13.3
HSI	3.7±1.4	3.9±1.4	3.8±1.4
FTND score	6.26±2.15	6.33±2.22	6.29±2.17

### SNP Selection and Genotyping

A venous blood specimen was obtained from each participant, and genomic DNA was extracted using a Qiagen Maxi kit (Qiagen Inc, Valencia, CA). Five and thirty-three SNPs were selected from the NCBI database (http://www.ncbi.nlm.nih.gov/projects/SNP/; build 128) for the long isoform of *GABBR1* and *GABBR2*, respectively. The SNPs selected in *GABBR2* were intended to provide more uniform coverage with less redundancy of those SNPs genotyped in our previous study [11]. Of the 33 SNPs in *GABBR2*, only two were genotyped for the second time (i.e., rs2304389 and rs3750344). The SNPs for both genes were chosen on the basis of minor allele frequency (≥0.10) and to obtain uniform physical coverage of the gene.

The SNPs for *GABBR1* were genotyped using a *Taq*Man assay in a 384-well microplate format, including four no-template negative controls and four positive controls for each homozygous genotype (12 total samples) (Applied Biosystems Inc., Foster City, CA) as described previously [11], [18]. Allelic discrimination was performed on an ABI Prism 7900HT Sequence Detection System (Applied Biosystems Inc., Foster City, CA). The *GABBR2* SNPs were genotyped using the Illumina BeadChip system at the Center for Inherited Disease Research (CIDR) at Johns Hopkins University.

### Individual SNP and Haplotype Analysis

First, we used Haploview (v. 4.0) [20] to identify any inconsistent Mendalian inheritance or other genotyping errors, revealing about a 0.04% error rate for *GABBR1* and 0.02% for *GABBR2*. These samples were excluded from further data analysis. Haploview (v. 4.0) was then used to assess linkage disequilibrium (LD) and haplotype blocks for all SNPs included in this study. Associations between individual SNPs and three ND measures were determined using the Pedigree-Based Association Test (PBAT; v. 3.5) [21]. Family-Based Association Test (FBAT; v. 1.7.3) software was used to determine associations between haplotypes and ND measures [22]. Haplotypes were determined using a sliding-window approach, where three consecutive SNPs were evaluated at once to determine the presence of any significant three-SNP combinations. Three genetic models (i.e., additive, dominant, and recessive) were tested for individual SNPs and haplotypes with sex and age as covariates; ethnicity was an additional covariate in the pooled sample, as all of these factors affect ND [2]. Results statistically significant at the 0.05 level for individual SNPs were determined by using a corrected *P* value of 0.002 for *GABBR2* and a corrected *P* value of 0.008 for *GABBR1* [23]. *P* values also were corrected for multiple testing of major haplotypes (frequency ≥0.05), which differed by SNP combination and sample grouping. We did not correct for three ND measure or genetic models because of the concern that they are highly related.

### Interaction Analysis of *GABBR1* and *GABBR2*

Gene-by-gene interactions between SNPs in *GABBR1* and *GABBR2* were analyzed using a newly developed PGMDR [24]. Selection of SNPs for interaction analysis for both genes was based on their physical location: SNPs located in exons that encode each functional domain of a subunit protein or SNPs within intronic regions bordered by exons encoding the transmembrane or cytoplasmic domain of each subunit. From *GABBR2*, we included the following SNPs: rs585819, rs669095, rs6478676, and rs13286336 on either side of exon 13, which encodes a transmembrane protein domain; rs2304389 and rs10985765 in exons 16 and 18, which encode two cytoplasmic protein domains, and rs785648 and rs10818739 in introns 16 and 18. From *GABBR1*, we included the following SNPs: rs29230 and rs29267 in exon 16 that encodes a transmembrane domain and intron 16, respectively, and rs2267633 in the 3′-UTR and rs2267635 in intron 6, which is bordered by exons that encode the extracellular domain.

As with the previous association analyses, age and sex were included as covariates for the EA and AA samples. Age, sex, and ethnicity were included as covariates in the pooled sample. Gene-by-gene interactions were examined for all two- to seven-locus models. The top-ranked interaction model was chosen for a given order of interaction, and its *P* value of prediction accuracy (PA) was evaluated by a permutation test based on 1000 shuffles of the adjusted phenotypic values. Because all *P* values reported here were based on permutation tests of each interaction model, no correction for multiple models is needed. For detailed information on the PGMDR approach, please refer to the paper by Lou et al. [24].

## Results

### Individual SNP Associations with ND for *GABBR1* and *GABBR2*

Table 2 shows allele frequencies (calculated by directly counting the number of each progenitor allele) and *P* values for those SNPs that showed significant associations with ND measures in at least one sample before correction for multiple testing. In addition to the seven *GABBR2* SNPs identified in our previous report [11], 11 unique SNPs were significantly positively associated with ND in the pooled sample (EA + AA; Table 2). However, the association of individual SNPs varied as a function of ethnicity. That is, each SNP identified as significant in the pooled sample was attributable to either the AA or the EA sample, and there were no SNPs with significant associations shared by the two ethnic groups. Specifically, SNPs rs10120452, rs12337255, rs2900512, and rs4743221 were associated with measures of ND in the AA sample, whereas SNPs rs2779543, rs6478676, and rs7865648 were associated with ND measures in the EA sample. Almost all SNPs were marginally associated with ND measures except for rs7865648 (*P* = 0.0009), which remained significantly associated with SQ in the EA sample after correction for multiple testing (Table 2). For *GABBR1*, analysis revealed no significant association with ND for any individual SNP in the three samples (data not shown); rs29230 exhibited a trend with FTND in the pooled sample (p = 0.08).

**Table 2:** Allele frequencies and *P* values for association of individual *GABBR2* SNPs with three measures of ND in pooled sample and for each ethnic group* †.

dbSNP ID (allele)	Pooled sample				AA sample				EA sample			
	MAF	SQ	HSI	FTND	MAF	SQ	HSI	FTND	MAF	SQ	HSI	FTND
**rs1930135** (C/T)	0.43	0.09a	0.06a	**0.04a**	0.48	0.24a	0.15a	0.07a	0.30	0.11a	0.12r	0.23r
**rs2779543** (G/A)	0.33	**0.04r**	**0.03d,r**	**0.02a**	0.38	0.12r	0.12r	0.06a	0.21	0.04d	0.02d	0.03d
**rs10120452** (G/A)	0.26	**0.008a**	**0.02a**	**0.01a**	0.30	**0.03a**	0.05a	**0.03a**	0.17	0.07a	0.07a	0.07a
**rs11788000** (T/C)	0.41	0.06a	0.06a	**0.04a**	0.44	0.18r	0.21r	0.08r	0.34	0.06a	0.05a	0.10a
**rs12337255** (T/C)	0.16	**0.07d**	0.07d	**0.04d**	0.14	**0.05d**	**0.03d**	**0.03d**	0.23	0.46r	0.64r	0.73d,r
**rs2900512** (C/T)	0.26	**0.03d,r**	**0.01d,r**	**0.006d,r**	0.28	0.06d	**0.02d**	**0.01d,r**	0.21	0.32d	0.26d	0.29d
**rs7044793** (G/T)	0.36	**0.03a**	**0.03d**	**0.03d,r**	0.36	0.07a	0.09r	0.12r	0.37	0.24r	0.12a	0.10a
**rs13295101** (T/C)	0.36	**0.04a,d,r**	**0.03d,r**	**0.03d,r**	0.36	0.08a	0.08r	0.11r	0.37	0.28r	0.16r	0.13r
**rs4743221** (A/C)	0.28	0.06r	0.08r	**0.02d,r**	0.30	0.06r	**0.04r**	**0.007d,r**	0.23	0.81d	0.59r	0.56r
**rs6478676** (G/A)	0.35	**0.02d,r**	**0.03r**	**0.03r**	0.32	0.22d	0.56d	0.40r	0.43	**0.008d,r**	**0.006d,r**	**0.006d,r**
**rs7865648** (C/T)	0.34	**0.006d**	**0.05r**	**0.04r**	0.23	0.60r	0.89a	0.82d	0.37	***0.0009r***	**0.01d,r**	**0.005r**

*Corrected *P* value at the 0.05 significance level is 0.002.

† Values significant before correction are shown in bold. Values that survive correction are shown in bold italics.

a  =  additive model, d  =  dominant model, r  =  recessive model.

**Figure 1:**
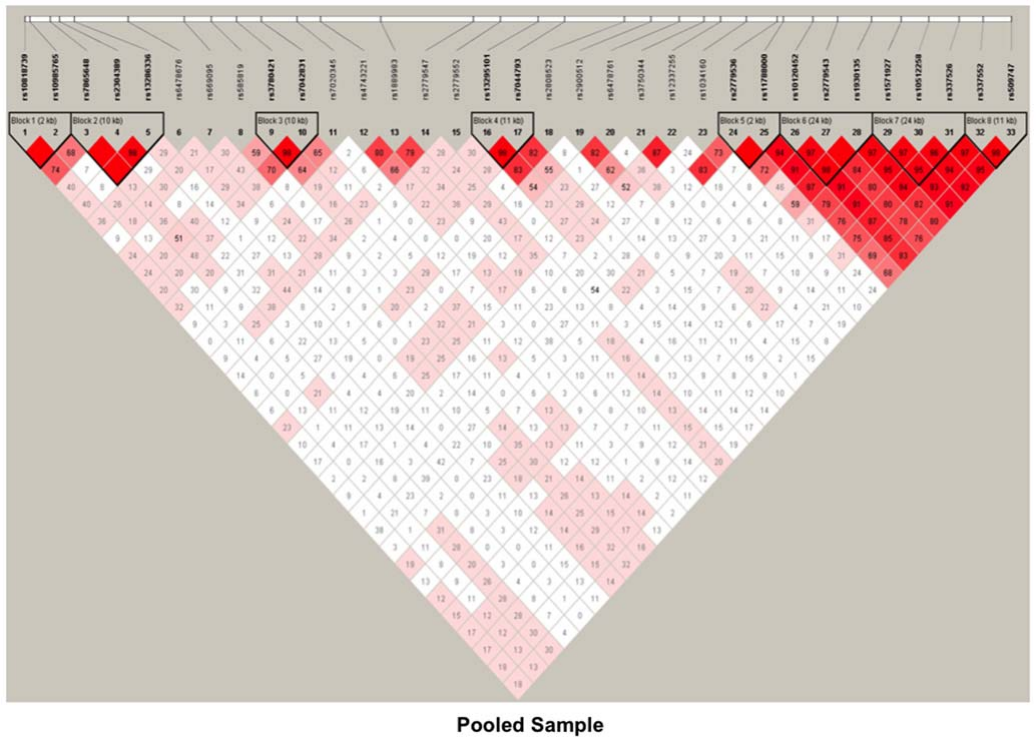
Linkage disequilibrium (LD) map of 33 SNPs within *GABBR2* in pooled sample. Dark red denotes high LD (D′ = 1 and LOD >2). Lower LD values are represented in shades of pink (0.21<D′<1 and LOD >2). White represents low LD and low LOD (LOD <2). Numbers indicate the D′ statistic for two consecutive SNPs. LD blocks were defined using Haploview [25].

**Figure 2:**
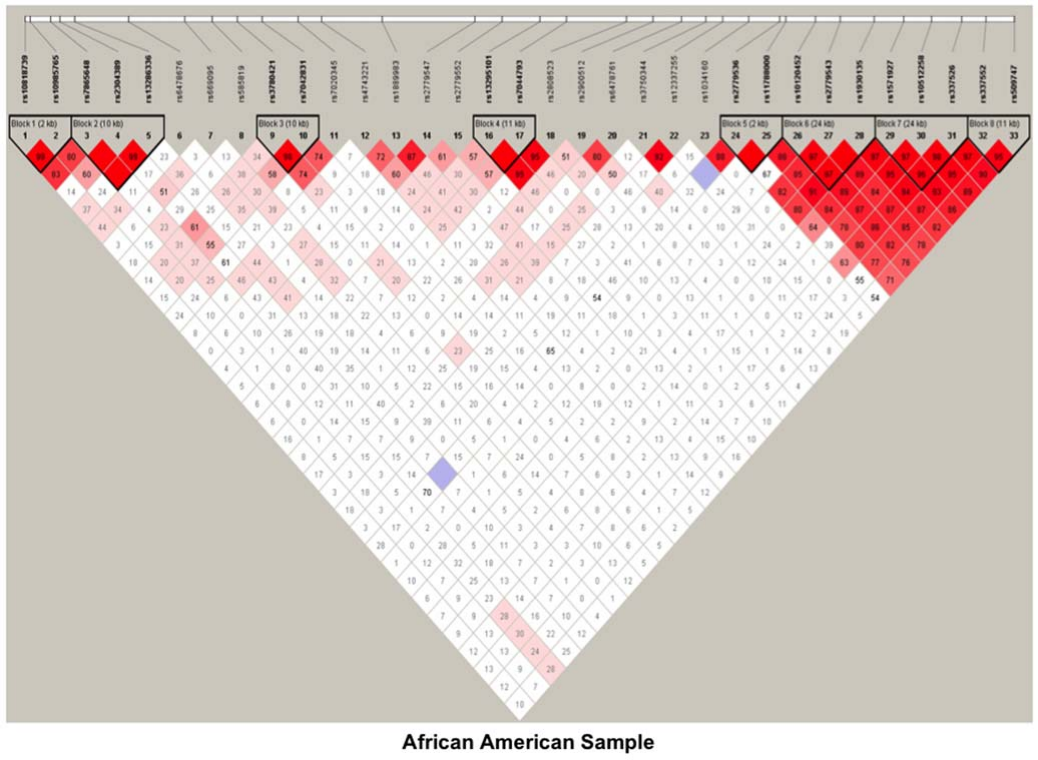
Linkage disequilibrium (LD) map of 33 SNPs within *GABBR2* in African American, sample. Dark red denotes high LD (D′ = 1 and LOD >2). Lower LD values are represented in shades of pink (0.21<D′<1 and LOD >2). White represents low LD and low LOD (LOD <2). Numbers indicate the D′ statistic for two consecutive SNPs. LD blocks were defined using Haploview [25].

**Figure 3:**
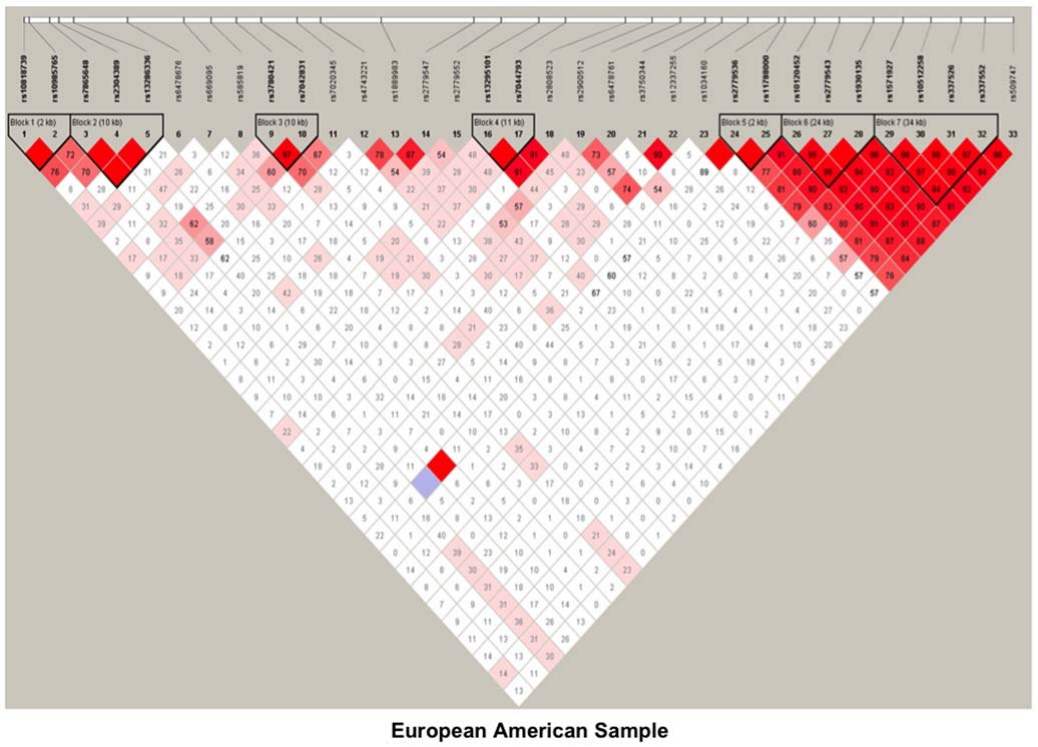
Linkage disequilibrium (LD) map of 33 SNPs within *GABBR2* in European American sample. Dark red denotes high LD (D′ = 1 and LOD >2). Lower LD values are represented in shades of pink (0.21<D′<1 and LOD >2). White represents low LD and low LOD (LOD <2). Numbers indicate the D′ statistic for two consecutive SNPs. LD blocks were defined using Haploview [25].

Using the block definition proposed by Gabriel et al. [25], we revealed eight blocks for *GABBR2* in the pooled sample (Figure 1). However, when LDs were assessed separately for each ethnic group, there were slight differences in LD blocks at the 5′ end of the gene. For example, we found four blocks at the 5′ end for AAs (Figure 2), and three blocks in the same region for EAs (Figure 3). Specifically, a 34-kb block in EAs containing SNPs rs157927-rs10512258-rs337526-rs337552 existed as two smaller blocks in AAs (rs157927-rs10512258-rs337526, 24 kb, and rs337552-rs509747, 11 kb).

**Figure 4:**
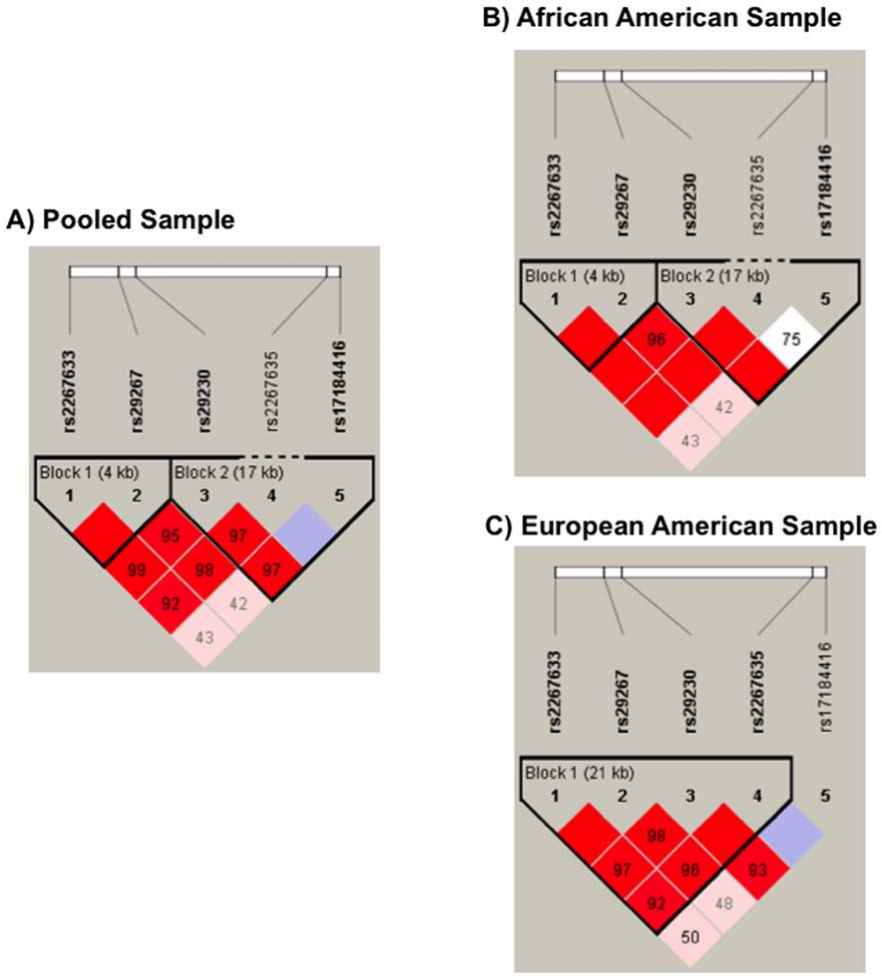
Linkage disequilibrium map of six SNPs within *GABBR1* in pooled, African-American, and European-American samples. Dark red denotes high linkage disequilibrium (LD) (D′ = 1 and LOD >2). Lower LD values are represented in shades of pink (0.21<D′<1 and LOD >2). White represents low LD and low LOD (LOD <2). Numbers indicate the D′ statistic for two consecutive SNPs. LD blocks were defined using Haploview [25].

Two blocks were identified in the pooled sample for *GABBR1* (Figure 4A); however, when parsed into different ethnic groups, the block structure became different in the two samples. The AA sample had two blocks spanning the whole gene, with the first 4-kb block containing rs2267633-rs29267 and a 17-kb block containing rs29230-rs2267635-rs17184416 (Figure 4B). Conversely, the EA sample had one 21-kb block consisting of SNPs rs2267633-rs29267-rs29230-rs2267635 located at the 3′ end of *GABBR1* (Figure 4C).

### Haplotype Associations with ND

**Figure 5:**
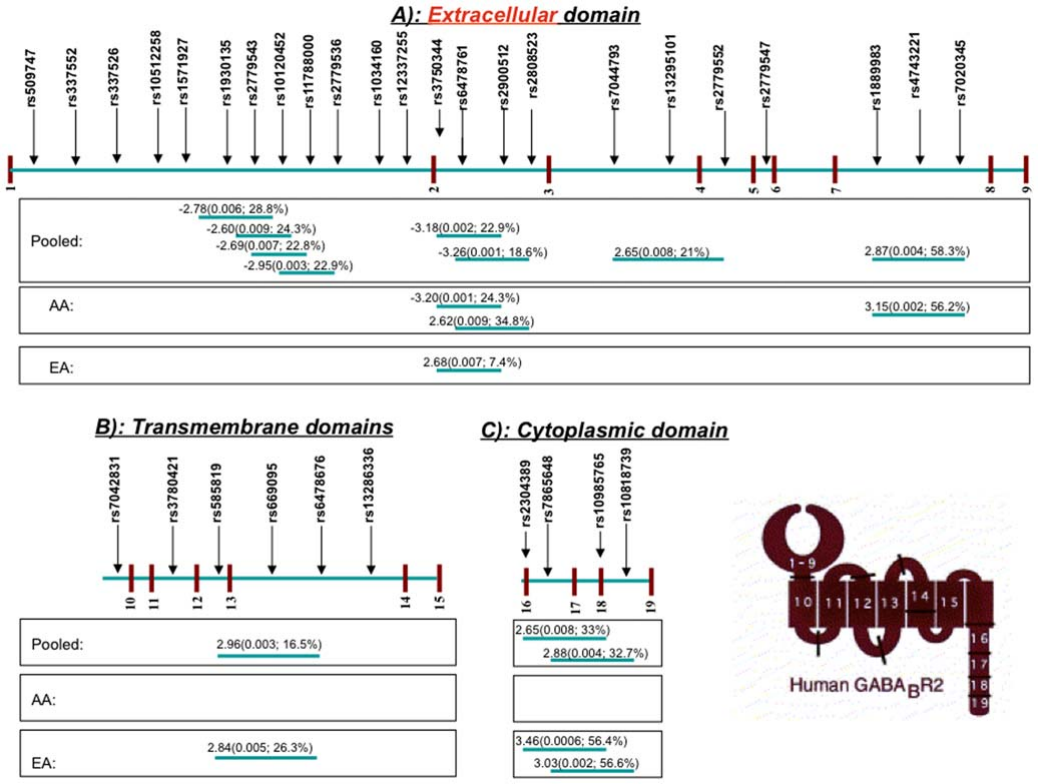
Physical location of detected significant major haplotypes on three functional domains of *GABBR2*. Vertical lines indicate exons; horizontal lines indicate introns in *GABBR2*. Arrows signify the physical location of SNPs within each protein domain. The configuration of the GABA_B2_ subunit protein is shown, containing numbers that correspond to the exons represented by vertical hash marks. Exons 1–9 are in the binding domain, exons 10–15 are in the transmembrane domains, and exons 16–19 are in the cytoplasmic domain of the receptor protein. Significant haplotypes are indicated for each sample group, including the *Z* score followed by *P* value and frequency in parentheses.

Haplotype-based association analyses revealed several major haplotypes that were significantly associated with ND for all sample groups. After correction for multiple testing, there were 14 significant major haplotypes in *GABBR2* in the pooled population (Table 3), four in the AA population (Table 4), and five in the EA population (Table 4). All the 14 haplotypes in the pooled sample were located within intronic areas among exons that encode the extracellular, transmembrane, and cytoplasmic domains, with most of them residing among extracellular domain encoding exons 1–9. When the two ethnic groups were analyzed separately, the haplotypes significantly associated with ND phenotypes differed greatly in the AA and EA populations (Table 4). In AAs, all four haplotypes that were significantly associated with FTND and one or two heaviness of smoking measures (HSI and SQ) resided in the intronic areas among extracellular domain-encoding exons (Table 4A and Figure 5A). Conversely, in EAs, four of the five significantly associated haplotypes were located in and around cytoplasmic and transmembrane domain coding exons 16–19 and 10–15, respectively (Tables 4B, 4C and Figures 5B, 5C). Only one haplotype located in the intronic areas among extracellular domain encoding exons was significantly associated with SQ, but not with FTND. Interestingly, the same three-SNP combination (i.e., rs3750344-rs6478761-rs2900512) constituted a haplotype in AAs with a differing allele combination, which was significantly associated with FTND and also HSI: The A-A-T haplotype (frequency 24.3%) was negatively associated with HSI (*Z* = −2.88, *P* = 0.004) and FTND (*Z* = −3.20, *P* = 0.001) in AAs, whereas a G-C-C haplotype (frequency 7.4%) was positively associated with SQ (*Z* = 2.68, *P* = 0.007) in EAs. Thus, our results clearly indicate that the significant haplotypes are ethnicity-specific, and the SNP loci of these haplotypes are located in very different parts of the gene in EAs and AAs. Another interesting phenomenon is that the direction of the effects of haplotypes was similar in the AA and EA populations if we did not consider the significance of haplotype effects, suggesting that even if the magnitude of their effects is a bit different in the various backgrounds, the allelic basis of ND in both groups is similar. In addition to the differences in haplotype locations in the gene, the directions of haplotype effects on FTND and heaviness of smoking (SQ and HSI) also differed in the two ethnic groups. As shown in Table 4, in AAs, the haplotypes significant under the recessive model were negatively associated with FTND, SQ, and HSI, whilst the two significant haplotypes under the dominant model were positively associated with FTND. Interestingly, these directions were reversed in EAs; all the significant haplotypes under the recessive model were positively associated with FTND, SQ, and HSI, and the dominant haplotype was negatively associated with SQ.

**Table 3:** Haplotype frequencies, and Z- and *P-* values for association of three-SNP combinations in *GABBR2* with three measures of ND in the pooled sample*.

SNP Combination	Haplotype	% (# Families)	*Z* value (*P*)		
			SQ	HSI	FTND
**(A) Extracellular Domain:**					
rs1571927-rs1930135-rs2779543	A-T-A	28.8 (82)	**−2.40 (0.017)r**	**−2.78 (0.006)r**	**−2.77 (0.006)r**
rs1930135-rs2779543-rs10120452	C-G-G	56.7(321.4)	2.16 (0.031)a	2.32 (0.020)a	**2.52 (0.012)a**
	T-A-A	24.3 (56)	−2.67 (0.023)r	**−2.60 (0.009)r**	**−2.50 (0.012)r**
rs2779543-rs10120452-rs11788000	G-G-T	57.7 (234.4)	2.27 (0.023)d	**2.53 (0.011)d**	**2.77 (0.006)d**
	A-A-C	22.8 (267.4)	**−2.69 (0.007)a**	**−2.63 (0.009)a**	**−2.62 (0.009)a**
rs10120452-rs11788000-rs2779536	G-T-A	59.1 (213.5)	2.26 (0.024)d	**2.58 (0.010)d**	**2.84 (0.005)d**
	A-C-A	22.9 (264.2)	**−2.95 (0.003)a**	**−2.68 (0.007)a**	**−2.67 (0.008)a**
rs3750344-rs6478761-rs2900512	A-A-T	22.9 (53)	−2.38 (0.017)r	**−2.96 (0.003)r**	**−3.18 (0.002)r**
rs6478761-rs2900512-rs2808523	A-C-C	28.2 (220.3)	1.98 (0.047)d	2.38 (0.017)d	2.62 (0.009)d
	A-T-C	18.6 (39)	**−3.26 (0.001)r**	**−3.20 (0.001)r**	**−3.17 (0.002)r**
rs7044793-rs13295101-rs2779552	T-C-G	21 (246.4)	**2.65 (0.008)a**	2.09 (0.037)a	1.99 (0.047)a
rs1889983-rs4743221-rs7020345	A-A-A	58.3 (218)	2.29 (0.021)d	2.37 (0.018)d	**2.87 (0.004)d**
**(B) Transmembrane Domain:**					
rs58519-rs669095-rs6478676	A-A-G	16.5 (21)	**2.96 (0.003)r**	2.48 (0.013)r	2.49 (0.013)r
**(C) Cytoplasmic Domain:**					
rs2304389-rs7865648-rs10985765	C-T-T	33 (84)	**2.65 (0.008)r**	1.94 (0.053)r	1.90 (0.058)r
rs7865648-rs10985765-rs10818739	T-T-A	32.7 (83)	**2.88 (0.004)r**	2.16 (0.031)r	2.17 (0.030)r

*Values shown in bold are significant after correction for multiple testing of major haplotypes (i.e., haplotypes with a frequency >5%). Corrected *P* values differ by population and haplotype. Models are the same as in Table 2.

**Table 4:** Haplotype frequencies, and Z- and *P-* values for association of three-SNP combinations in *GABBR2* with three measures of ND in the AA and EA populations*.

SNP Combination	Haplotype	AA sample				EA sample			
			Z value (P)				Z value (P)		
		% (# Families)	SQ	HSI	FTND	% (# Families)	SQ	HSI	FTND
**(A) Extracellular Domain:**									
rs1571927-rs1930135-rs2779543	A-T-A	36.4(78)	−1.93(0.054)r	−2.267(0.024)r	−2.38(0.018)r	14.1(53.8)	−1.55(0.120)a	−1.58(0.115)a	−1.47(0.141)a
rs1930135-rs2779543-rs10120452	C-G-G	48.7(239.3)	1.58(0.113)a	1.83(0.067)a	2.23(0.026)a	71.8(32)	1.65(0.098)d	1.62(0.105)d	1.32(0.185)d
	T-A-A	29.5(52)	N/A	−2.12(0.034)r	−2.12(0.034)r	14.5(53)	−1.75(0.080)a	−1.85(0.064)a	−1.86(0.063)a
rs2779543-rs10120452-rs11788000	G-G-T	52.4(194.4)	1.79(0.073)d	2.02(0.043)d	2.42(0.016)d	67.7(87)	1.78(0.075)a	1.78(0.075)a	1.56(0.118)a
	A-A-C	27.3(211.5)	−2.16(0.030)a	−2.05(0.040)a	−2.05(0.040)a	14.5(53)	−1.75(0.080)a	−1.91(0.056)a	−1.88(0.060)a
rs10120452-rs11788000-rs2779536	G-T-A	54.2(182.5)	1.71(0.087)d	2.03(0.043)d	2.46(0.014)d	68(84)	1.87(0.061)a	1.83(0.067)a	1.63(0.103)a
	A-C-A	27.2(208)	−2.38(0.018)a	−2.04(0.042)a	−2.03(0.042)a	14.8(53)	−1.89(0.059)a	−2.04(0.041)a	−2.03(0.042)a
rs3750344-rs6478761-rs2900512	A-A-T	24.3(39)	−2.22(0.026)r	**−2.88(0.004)r**	**−3.20(0.001)r**	19.9(14)	−0.98(0.329)r	−0.96(0.336)r	N/A
	G-C-C	8(83.9)	−0.22(0.829)a	−0.74(0.458)a	−0.99(0.320)a	7.4(28.2)	**2.68(0.007)d**	1.93(0.054)d	2.07(0.039)d
rs6478761-rs2900512-rs2808523	A-C-C	34.8(175)	1.99(0.047)d	2.37(0.018)d	**2.62(0.009)d**	15(43.4)	N/A	0.46(0.643)d	0.39(0.695)d
	A-T-C	24.4(39)	**−3.30(0.0009)r**	**−3.25(0.001)r**	**−3.24(0.001)r**	6.9(32.5)	−1.04(0.299)d	−0.48(0.634)d	−0.51(0.612)d
rs7044793-rs13295101- rs2779552	T-C-G	17.1(165)	2.11(0.035)a	1.28(0.199)a	N/A	29.5(83.4)	1.41(0.159)a	1.66(0.097)a	1.60(0.110)a
rs1889983-rs4743221-rs7020345	A-A-A	56.2(171.2)	2.15(0.031)d	2.47(0.014)d	**3.15(0.001)d**	62(43.2)	1.10(0.273)d	0.74(0.460)d	0.58(0.561)d
**(B) Transmembrane Domain:**									
rs58519-rs669095-rs6478676	A-A-G	11(116.4)	0.22(0.827)a	0.27(0.784)a	N/A	26.3(14)	**2.84(0.005)r**	**2.51(0.012)r**	**2.63(0.009)r**
**(C) Cytoplasmic Domain:**									
rs2304389-rs7865648-rs10985765	C-T-T	20.5(37)	N/A	−0.29(0.774)r	−0.50(0.618)r	56.4(48)	**3.46(0.0006)r**	**2.88(0.004)r**	**3.13(0.002)r**
rs7865648-rs10985765-rs10818739	T-T-A	20.2(174.8)	N/A	−0.39(0.694)d	−0.66(0.510)d	56.6(47)	**3.38(0.0007)r**	**2.78(0.005)r**	**3.03(0.002)r**
	C-T-A	27.2(44)	−0.68(0.495)r	−0.83(0.408)r	−0.99(0.324)r	23.5(64.8)	**−2.70(0.007)d**	−2.09(0.037)d	−2.38(0.017)d

*Values shown in bold are significant after correction for multiple testing of major haplotypes (i.e., haplotypes with a frequency >5%). Corrected *P* values differ by population and haplotype. Models are the same as in Table 2.

Haplotype analyses of *GABBR1* revealed no major haplotypes associated significantly with ND (data not shown). There were, however, two minor haplotypes in the EA population (frequency <5%) that were positively associated with HSI after correction for multiple testing: rs2267635-rs29230-rs29267 (C-C-T; frequency 3.6%; *Z* = 2.7, *P* = 0.007) and rs29230-rs29267-rs2267633 (C-T-A; frequency 3.8%; *Z* = 2.7, *P* = 0.007) (Table 5), both consisting of SNP rs29230, a synonymous SNP in exon 16 encoding a transmembrane domain protein. Although the SNPs selected for this gene were targeted at the *GABBR1* long isoform, these four SNPs also exist in the short isoforms of *GABBR1.*

**Table 5:** Haplotype frequencies, and Z- and *P-*values for association of three-SNP combinations in *GABBR1* with three measures of ND in EA population* †.

SNP Combination	Haplotype	% (# Families)	*Z*-value (*P*)		
			SQ	HSI	FTND
rs2267635-rs29230- rs29267	C-T-T	8.3 (55)	−1.30 (0.192)r	−2.02 (0.044)r	−2.07 (0.039)r
	C-C-T	3.6 (22)	2.31 (0.020)a,d	**2.72 (0.007)a,d**	2.40 (0.017)a,d
rs29230- rs29267- rs2267633	T-T-A	8.4 (55)	−1.30 (0.193)r	−2.02 (0.044)r	−2.07 (0.039)r
	C-T-A	3.8 (22)	2.31 (0.021)a,d	**2.72 (0.007)a,d**	2.40 (0.017)a

*Values shown in bold are significant after correction for multiple testing of major haplotypes (i.e., haplotypes with a frequency >5%). Corrected *P* values differ by population and haplotype. Models are the same as in Table 2.

† Note that significant haplotypes are minor haplotypes (i.e.,<5%).

### Interaction Analysis of *GABBR1* and *GABBR2*

On the basis of the knowledge obtained from biochemical and pharmacological studies of the GABA_B_ receptor, we investigated the epistatic effect of SNPs from the transmembrane and cytoplasmic domains of each GABA_B_ subunit protein because interactions among these domains have biological significance (Figure S1). That is, these regions of the receptor proteins are involved in coupling of the subunits for trafficking to the membrane and for pharmacological activity. Our gene-by-gene interaction analysis revealed significant epistatic effects between *GABBR1* and *GABBR2* (Table 6) on ND in the pooled and EA samples and also interactions among SNPs within *GABBR2* in all three samples (Table S1).

**Table 6:** Detected interaction models for SNPs in *GABBR1* and *GABBR2.*

Sample	Gene(s) and SNPs included in interaction model	ND measure	Prediction accuracy	Permutated *P* value
Pooled	*GABBR1*: rs29230	HSI/FTND	0.55	**0.001**
	*GABBR2*: rs7865648-rs585819	HSI/FTND	0.54	**0.005**
	*GABBR1*: rs29230; *GABBR2*: rs7865648-rs669095-rs585819	FTND	0.52	**0.05**
EA	*GABBR1*: rs29230; *GABBR2*: rs7865648-rs6478676-rs585819	FTND	0.56	**0.02**

Three significant interactions were detected between SNPs in *GABBR1* and *GABBR2*. The same three-SNP combination was detected for HSI and FTND (rs29230-rs7865648-rs585819; *P* = 0.001 and 0.005, respectively), and the addition of a fourth SNP to this combination was detected for FTND (rs29230-rs7865648-rs669095-rs585819; *P* = 0.05). This four-SNP combination was contributed by the EA population for FTND (*P* = 0.02), whereas the other interactions were detected only in the pooled sample.

The majority of significant SNP interactions were seen within *GABBR2* for the pooled, EA, and AA samples (Table S1). Five significant two- to four-SNP interactions were detected in the pooled sample for SQ (rs10985765-rs7865648, *P*<0.001; rs10985765-rs7865648-rs6478676, *P* = 0.003; and rs10818739-rs7865648-rs6478676-rs585819, *P* = 0.004) and HSI (rs10985765-rs13286336-rs669095-rs585819, *P* = 0.05; and rs10985765-rs7865648-rs6478676-rs669095, *P* = 0.03). In the AA population, one four-SNP combination, rs10985765-rs13286336-rs699095-rs585819, was significantly associated with all three ND measures (*P* = 0.01–0.02). All other significant two- to four-SNP interactions were identified in the EA sample: SQ and FTND: rs7865648-rs585819, *P*<0.001; rs10985765-rs7865648-rs6478676-rs669095, *P* = 0.003; and 0.01; SQ alone: rs10818739-rs7865648-rs669095, *P*<0.001 and HSI: rs10818739-rs585819, *P* = 0.003; rs10818739-rs6478676-rs585819, *P*<0.001; and rs10818739-rs7865648-rs6478676-rs585819, *P* = 0.03 (Table S1).

## Discussion

The pharmacologically active GABA_B_ receptors are formed by heterodimerization of GABA_B1_ and GABA_B2_ subunit proteins encoded by *GABBR1* and *GABBR2* genes, respectively. The current study examined the association of *GABBR1* and *GABBR2* polymorphisms with ND individually and according to their interactive effects in European-American and African-American individuals.

First, our findings revealed a significant association of heaviness of smoking with SNP rs7865648 in EAs and significant associations of ND with 14 major haplotypes in *GABBR2* in the pooled sample, after correction for multiple testing. When SNP data were analyzed within each ethnic group, we found two protective and two risk haplotypes unique to the AA sample and one protective and four risk haplotypes unique to the EA sample. It was striking that these unique loci were distributed in different regions of the gene in the two races. Only one common SNP combination was significant in the two ethnic samples but with a differing allelic haplotype in each population, EAs possessing a risk haplotype (G-C-C) and AAs having a protective haplotype (A-A-T). The five polymorphisms in *GABBR1* studied here showed no significant associations with any of the ND measures; the smallest *P* value for association of rs29230 with FTND was 0.08 in the pooled sample. Haplotype analyses revealed two minor haplotypes that were significantly associated with HSI but not with FTND in the EA sample.

Second, in consideration of the fact that functional GABA_B_ receptors consist of both GABA_B1_ and GABA_B2_ subunits, we conducted gene-gene interaction analysis of these two subunit genes in affecting ND. Analyses using our newly developed PGMDR method [24] demonstrated a significant interaction between *GABBR2* and *GABBR1* polymorphisms, confirming previous findings of pharmacological studies that showed GABA_B_ receptors function as heterodimers of GABA_B1_ and GABA_B2_ subunits. Together, our results provide first evidence for direct association of ND with *GABBR2* polymorphisms and an indirect less significant association with *GABBR1* polymorphisms.

Our current study has several unique strengths compared with previous studies from our group and others that implicated loci on chromosome 9, including the *GABBR2* gene, in altering susceptibility to ND [4], [16]. On the basis of our early linkage findings, in a previous study, we genotyped SNPs in *GABBR2* and found that seven of the 12 SNPs initially identified were significantly associated with ND [11]. In consideration of the relatively scanty coverage of *GABBR2* and the small sample size in our earlier study, we conducted the current study with the goal of extending our previous results. In the previous study, 12 SNPs were genotyped in about half of the participants of the current MSTF sample. Also, we chose SNPs that provided complete coverage of the gene, without consideration of previously genotyped SNPs. Thus, the current study extends the previous findings by: (a) comprehensively covering the gene by genotyping 33 SNPs for *GABBR2*, (b) increasing our sample size by ∼760 participants, (c) including an association analysis of *GABBR1* with ND, and (d) examining the interaction between *GABBR1* and *GABBR2*.

The GABA_B1_ and GABA_B2_ subunits dimerize by coiling of C-terminal domains of the two proteins following which the subunits are transported to the plasma membrane, where the receptor becomes pharmacologically active [26], [27]. Further evidence suggests the involvement of transmembrane domains in the formation of heterodimers when the C-terminal domain is truncated [28]. This is particularly interesting, considering that, in EAs, haplotypes significantly associated with ND reside in the introns among the transmembrane and cytoplasmic-domain-encoding exons of *GABBR2*. Conversely, all haplotypes implicated in ND of AAs reside in the introns among exons encoding the binding domain of the *GABBR2* subunit protein. The function of the binding domain of GABA_B2_ is unclear, however, as only GABA_B1_ is responsible for ligand binding in the heterodimer configuration [29]. Therefore, haplotypes that affect different portions of the GABA_B_ heterodimer may have functionally different consequences. For example, EAs may possess GABA_B2_ subunits that are unable to dimerize with the GABA_B1_ subunit, which consequently cannot traffic to the membrane, resulting in fewer functional receptors. Conversely, the affected GABA_B2_ binding domain has not been fully characterized, so it is difficult to speculate on the consequences of these polymorphisms.

Although we found significant associations for *GABBR2*, but not for *GABBR1*, with ND, significant interactions between these two subunit genes were evident as reported here. Significant interactions were detected between a synonymous SNP in the transmembrane domain of *GABBR1* and SNPs located in the intronic regions among exons encoding transmembrane and cytoplasmic domains of *GABBR2*. These statistical gene-by-gene interactions are biologically relevant, as the subunits interact to form a complete and functional receptor. Thus, the statistical interaction most likely represents the functional properties of these two subunits. Furthermore, we found that the majority of significant interactions exist within the *GABBR2* gene, suggesting a stronger association of ND with *GABBR2* polymorphisms compared to associations of ND with both *GABBR1* polymorphisms and *GABBR1*-by-*GABBR2* interactive effects. However, it should also be noted that the *GABBR2* SNPs included in the interaction models are located in the intronic regions among exons encoding transmembrane and cytoplasmic domains of the GABA_B2_ subunit. Therefore, these polymorphisms do not affect the amino acid sequence of the transmembrane and cytoplasmic subunits unless there is a strong linkage disequilibrium with a causative variant in an exon; nevertheless, it is possible they affect the structure of mature GABA_B2_ mRNA through alternate splicing, resulting in altered GABA_B2_ protein subunits. Although such a molecular mechanism is yet to be elaborated, the presence of six alternatively spliced mRNA variants for GABA_B2_ (http://www.ncbi.nlm.nih.gov/IEB/Research/Acembly/) strengthens the significance of functional SNPs in *GABBR2* intronic regions.

In sum, this study not only confirms our earlier finding that *GABBR2* is associated with ND but also demonstrates that an interaction of *GABBR1* and *GABBR2* alters susceptibility to ND. Our findings also indicated that *GABBR2* represents a major contributor to this phenomenon, and its biological and statistical interaction with *GABBR1* implies that the latter is indirectly involved in ND. Furthermore, our results showed that the genetically determined vulnerability to ND is different in subjects of European and African ancestry. These findings are further supported by studies that demonstrated the regulation of receptor expression after nicotine exposure in animal models, as well as the impact on addictive behaviors in both animals and humans of the GABA_B_ agonist balcofen.

## Supporting Information

Table S1 Supplementary Table S1
(0.04 MB DOC)

Figure S1 Supplementary Figure 1
(3.00 MB TIF)
