# Supplementary material for: Association and Interaction Analyses of GABBR1 and GABBR2 with Nicotine Dependence in European- and African-American Populations
Source: PLoS One. 2009 Sep 18;4(9):e7055. doi: 10.1371/journal.pone.0007055 (PMC2739294; doi:10.1371/journal.pone.0007055)
Supplement: Table S1 — Supplementary Table S1 (0.04 MB DOC) [file pone.0007055.s001.doc]

**Table S1**. Detected interaction models for genotype combinations formed by SNPs in *GABBR2*

| Sample | SNPs included in interaction model | ND  measure | Prediction  accuracy | Permutated  *P* value | Functional  domain(s) |
| --- | --- | --- | --- | --- | --- |
| Pooled | rs10985765-rs7865648 | SQ | 0.54 | **<0.001** | Cytoplasmic |
|  | rs10985765-rs7865648-rs6478676 | SQ | 0.54 | **0.003** | Cytoplasmic and Transmembrane |
|  | rs10818739-rs7865648-rs6478676-rs585819 | SQ | 0.52 | **0.004** |  |
|  | rs10985765-rs13286336-rs669095-rs585819 | HSI | 0.52 | **0.05** |  |
|  | rs10985765-rs7865648-rs6478676-rs669095 | HSI | 0.53 | **0.03** |  |
| AA | rs10985765-rs13286336-rs669095-rs585819 | SQ/ | 0.53 | **0.02** | Cytoplasmic and Transmembrane |
|  |  | HSI/ | 0.53 | **0.02** |  |
|  |  | FTND | 0.54 | **0.01** |  |
| EA | rs10818739-rs585819 | HSI | 0.54 | **0.003** | Cytoplasmic and Transmembrane |
|  | rs7865648-rs585819 | HSI/ | 0.60 | **<0.001** |  |
|  |  | FTND | 0.58 | **<0.001** |  |
|  | rs10818739-rs7865648-rs669095 | SQ | 0.61 | **<0.001** |  |
|  | rs10818739-rs6478676-rs585819 | HSI | 0.55 | **<0.001** |  |
|  | rs10818739-rs7865648-rs6478676-rs585819 | HSI | 0.50 | **0.03** |  |
|  | rs10985765-rs7865648-rs6478676-rs669095 | SQ/ | 0.56 | **0.003** |  |
|  |  | FTND | 0.56 | **0.01** |  |
