# Supplementary figures and images for: Association and Interaction Analyses of GABBR1 and GABBR2 with Nicotine Dependence in European- and African-American Populations
Source: PLoS One. 2009 Sep 18;4(9):e7055. doi: 10.1371/journal.pone.0007055 (PMC2739294; doi:10.1371/journal.pone.0007055)

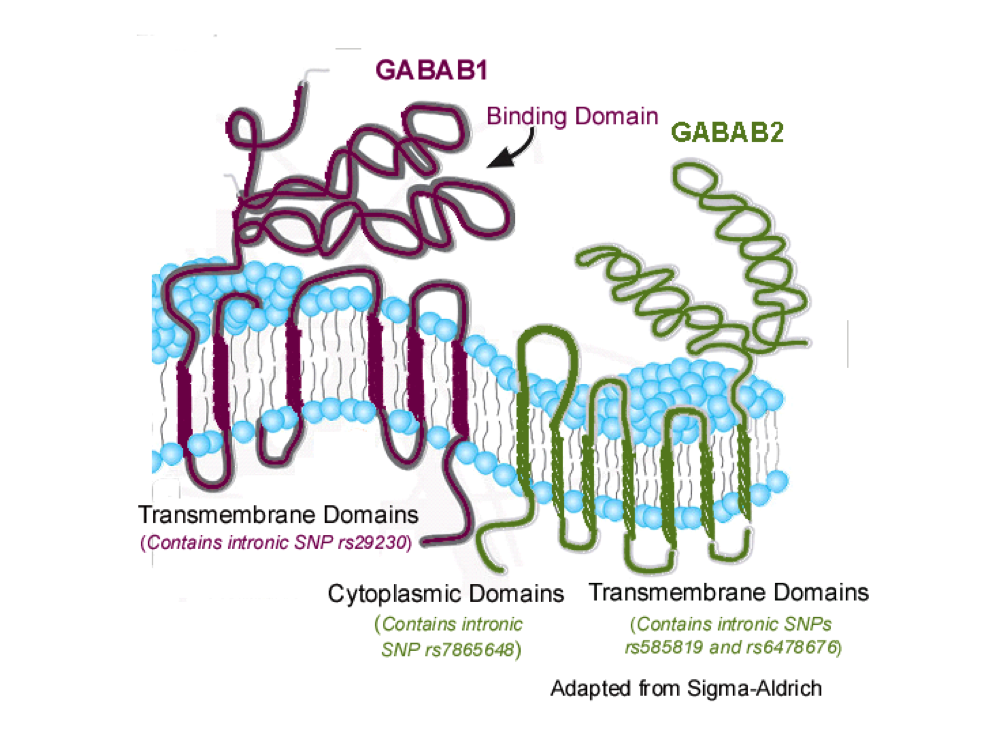

Supplement: Figure S1 — Supplementary Figure 1 (3.00 MB TIF) [file pone.0007055.s002.tif]
